# Evaluation of the U.S. EPA/OSWER Preliminary Remediation Goal for Perchlorate in Groundwater: Focus on Exposure to Nursing Infants

**DOI:** 10.1289/ehp.9533

**Published:** 2006-12-11

**Authors:** Gary L. Ginsberg, Dale B. Hattis, R. Thomas Zoeller, Deborah C. Rice

**Affiliations:** 1 Connecticut Department of Public Health, Hartford, Connecticut, USA; 2 Clark University, Worcester, Massachusetts, USA; 3 University of Massachusetts, Amherst, Massachusetts, USA; 4 Maine Center for Disease Control and Prevention, Augusta, Maine, USA

**Keywords:** drinking water, neurodevelopment, nursing infants, perchlorate, PRG, thyroid hormone

## Abstract

**Background:**

Perchlorate is a common contaminant of drinking water and food. It competes with iodide for uptake into the thyroid, thus interfering with thyroid hormone production. The U.S. Environmental Protection Agency’s Office of Solid Waste and Emergency Response (OSWER) set a groundwater preliminary remediation goal (PRG) of 24.5 μg/L to prevent exposure of pregnant women that would affect the fetus. This does not account for the greater exposure that is possible in nursing infants or for the relative source contribution (RSC), a factor normally used to lower the PRG due to nonwater exposures.

**Objectives:**

Our goal was to assess whether the OSWER PRG protects infants against exposures from breast-feeding, and to evaluate the perchlorate RSC.

**Methods:**

We used Monte Carlo analysis to simulate nursing infant exposures associated with the OSWER PRG when combined with background perchlorate.

**Results:**

The PRG can lead to a 7-fold increase in breast milk concentration, causing 90% of nursing infants to exceed the reference dose (RfD) (average exceedance, 2.8-fold). Drinking-water perchlorate must be < 6.9 μg/L to keep the median, and < 1.3 μg/L to keep the 90th-percentile nursing infant exposure below the RfD. This is 3.6- to 19-fold below the PRG. Analysis of biomonitoring data suggests an RSC of 0.7 for pregnant women and of 0.2 for nursing infants. Recent data from the Centers for Disease Control and Prevention (CDC) suggest that the RfD itself needs to be reevaluated because of hormonal effects in the general population.

**Conclusions:**

The OSWER PRG for perchlorate can be improved by considering infant exposures, by incorporating an RSC, and by being responsive to any changes in the RfD resulting from the new CDC data.

Perchlorate is a powerful oxidant that is used in rocket fuel, munitions, blasting operations, and fireworks [National Research Council (NRC) 2005]. Environmental contamination has occurred at military installations, at facilities that make perchlorate, and at various construction sites from the blasting of bedrock to build roads or homes. In addition, there are natural sources of perchlorate such as fertilizer produced in certain regions (e.g., Chilean nitrate), evaporite soils, and atmospheric sources (Dasgupta et al. 2005; Orris et al. 2003). Its high water solubility and environmental persistence have led to contamination of groundwater, with detection increasing in recent years as analytical methods have improved [Government Accountability Office (GAO) 2005]. There are no federal drinking water standards for perchlorate, although a number of states have recently developed or proposed values in the 2–6 μg/L range [Massachusetts Department of Environmental Protection (MADEP) 2006; New Jersey Drinking Water Quality Institute 2005; Ting et al. 2006]. These drinking-water targets are intended to prevent perchlorate’s neurodevelopmental effects resulting from its antithyroid action.

Perchlorate can impair thyroid function by inhibiting the uptake of iodide, thereby reducing the amount of iodide stored in the thyroid and available for hormone production (NRC 2005; Ting et al. 2006). In those who have adequate iodide intake and stores of thyroid hormone, this impairment can be overcome with little to no consequence (Braverman et al. 2005). However, gestation can be a vulnerable period because the mother has increased nutritional demands for iodide and because thyroid hormone is critically important for fetal brain development (NRC 2005). The U.S. Environmental Protection Agency (EPA) reference dose (RfD) of 0.0007 mg/kg/day, as adopted from a report from the National Research Council (NRC 2005; U.S. EPA 2005), is intended to protect the general public, including vulnerable life stages such as *in utero* development, from perchlorate’s antithyroid effects. This RfD has been used in at least one case to derive a drinking-water limit for perchlorate (New Jersey Drinking Water Quality Institute 2005), whereas other states have used more stringent toxicity values to set a drinking-water limit (MADEP 2006; Ting et al. 2006). The case for a lower RfD has also been made by others (Ginsberg and Rice 2005). Recent data from the Centers for Disease Control and Prevention (CDC) indicate a low-dose effect of perchlorate, particularly on women with low iodine intake, and thus suggest a need to lower the RfD (Blount et al. 2006a).

In the present article we do not focus on the issue of the appropriateness of the U.S. EPA RfD, but rather evaluate whether a groundwater cleanup guideline issued by U.S. EPA’s Office of Solid Waste and Emergency Response (OSWER) would keep exposure below the RfD for all vulnerable segments of the population. The OSWER guideline, released January 2006, sets a groundwater preliminary remediation goal (PRG) of 24.5 μg/L for Superfund sites containing perchlorate. Whereas this level corresponds to the amount that would deliver the RfD for a 70-kg adult ingesting 2 L/day, it is not necessarily protective of nursing and bottle-fed infants who consume more liquid per body weight than adults (U.S. EPA 2002). A recent analysis calculated perchlorate doses that were above the RfD for infants drinking reconstituted formula made with water containing perchlorate at 24 μg/L, the OSWER PRG (Baier-Anderson et al. 2006). Further, from a limited breast milk biomonitoring data set, Kirk et al. (2005) estimated that nursing infants could receive doses above the RfD even without considering the added exposure associated with the OSWER PRG.

Our primary objective is to evaluate the perchlorate dose to nursing infants resulting from maternal ingestion of water contaminated by perchlorate at the OSWER PRG of 24.5 μg/L. As explained below and described elsewhere (Baier-Anderson et al. 2006), infants are likely also to be highly susceptible to perchlorate. The OSWER PRG did not explicitly consider exposure during this life stage.

An additional objective is to evaluate whether the OSWER PRG protects the pregnant mother and her developing fetus. Exposure to the fetus depends on the mother’s intake of perchlorate from both diet and drinking water. In setting drinking-water maximum contaminant levels (MCLs), the U.S. EPA routinely applies a relative source contribution (RSC) to allow for the possibility that not all exposure will come from water, recognizing the importance of keeping the total exposure dose (e.g., water plus diet) below the RfD. The default RSC is 0.2, meaning that only 20% of the RfD would be allowed to come from drinking water. In the case of the OSWER PRG for perchlorate, the groundwater target is set at the water concentration that corresponds to the RfD—in effect, setting the RSC to unity. This appears to be contrary to the emerging database on perchlorate content of foods, which shows that perchlorate is common in the diet [El Aribi et al. 2006; U.S. Food and Drug Administration (FDA) 2004]. The limited human biomonitoring data suggest widespread exposure, with dietary perchlorate appearing to be a key source (Kirk et al. 2005; Valentin-Blasini et al. 2005). This indicates a need for careful consideration of the RSC. We provide a means to do this by analyzing the available human biomonitoring data.

Some may be less concerned about exceedance of the RfD because it is based on a precursor effect, inhibition of iodide uptake by the thyroid. This implies that the RfD prevents a biochemical change that precedes a more serious toxic effect, and thus is not itself a critical health end point. This assumption lacks support; there are no data that show how much iodide uptake inhibition is needed to affect thyroid function. This relationship is likely to depend on a number of host-specific factors. For example, recent observations by Blount et al. (2006a) demonstrate that women in the lowest category of iodine intake were most sensitive to perchlorate’s effects on thyroid hormone production. Analogous to the low iodine women in the Blount et al. study (2006a), neonates are likely to be a sensitive life stage because of perchlorate’s direct effects on the thyroid and its ability to limit iodine transfer into breast milk, thereby reducing infant intake of this nutrient (Kirk et al. 2005; Tellez et al. 2005). Moreover, the simultaneous exposure to other breast milk contaminants (e.g., polychlorinated biphenyls, polybrominated diphenyl ethers, dioxins) that can disrupt thyroid function by other modes of action may interact with perchlorate in infants. Therefore, limiting perchlorate exposure should be a critical public health target not only during pregnancy but also in infants. This rationale is further described below.

## Literature Review: Why Focus on Perchlorate Effects in Infants?

If the perchlorate mechanism of action is not relevant to the postnatal period, or if this period is considerably less sensitive than the *in utero* period, then application of the RfD to this period would be inappropriate. Therefore, this analysis begins with a literature review describing factors that may affect susceptibility to perchlorate during the post-natal period. Because there is no indication that the perchlorate mechanism of action should differ across life stages, our review focuses on the ability of neonates to compensate for perchlorate-induced decreases in thyroid hormone synthesis.

### Sensitivity of newborns to thyroid disruption and altered brain development

During the *in utero* period, the fetal brain undergoes critical developmental stages that are supported by the maternal supply of thyroid hormone T_4_ (thyroxine) (Howdeshell 2002). Maternal T_4_ is an important source of thyroid hormone for the fetus throughout gestation. It is the only source during the first trimester (Howdeshell 2002; Morreale de Escobar 2001), and remains an important complement during late gestation, when it contributes approximately 30% to the fetal supply of T_4_ (Vulsma et al. 1989)

The importance of maternal T_4_ has been demonstrated in babies with congenital hypothyroidism who appear normal at birth because of ample maternal hormone during gestation (Vulsma et al. 1989). In contrast to the fetus, the newborn can no longer rely on maternal hormone as a buffer against inborn biosynthetic deficiencies or external stressors. The only means for hormone transfer from the mother is breast milk; however, breast milk contains very little thyroid hormone (van Wassenaer et al. 2002). Therefore, the neonate must synthesize its own supply of T_4_ to maintain normal growth and development. As described below, several factors make neonatal thyroid status more vulnerable to perturbation than in adults or the fetus.

First, the serum half-life of T_4_ is approximately 7–10 days in adults (Chopra and Sabatino 2000), but is approximately 3 days in neonates (Lewander et al. 1989; van den Hove et al. 1999). Thus, the rate of replacement of T_4_ (i.e., T_4_ secretion from the thyroid gland) must be considerably higher in early life to maintain steady-state levels. Second, the adult thyroid gland stores a large quantity of thyroid hormone in the form of thyroglobulin; this quantity is estimated to be enough to maintain normal levels of circulating hormone for several months (Greer et al. 2002). In contrast, the neonatal gland stores very little T_4_; the amount stored has been estimated at less than that required for a single day (Savin et al. 2003; van den Hove et al. 1999). These differences indicate that the functional reserve available to adults is virtually absent in neonates. Any reduction in thyroid hormone synthesis in the neonate will result in a reduction in circulating levels, whereas this is clearly not true for the adult. The combined storage deficiency and rapid hormone turnover in neonates necessitates a high rate of T_4_ synthesis to keep up with the daily demand for thyroid hormone. This, in turn, depends on an adequate supply of iodide. Given these demands on the neonatal thyroid, it is likely that perchlorate-induced inhibition of iodide uptake has a greater impact in neonates than *in utero* or at other life stages. This is consistent with a recent study of 2,3,7,8-tetrachlorodibenzo-*p*-dioxin (TCDD) in rats that showed that post-natal (lactational) exposure produced greater thyroid disruption than exposure during the *in utero* period (Nishimura et al. 2005). Although rats have a different developmental time frame than humans, and TCDD’s mechanism of thyroid disruption differs from that of perchlorate, the rat findings suggest an important postnatal window of vulnerability to thyroid toxicants. The concern for post-natal effects is magnified with perchlorate because of its potential to also interfere with iodide excretion into breast milk.

Considering these factors, it is critical to understand the degree to which iodide uptake must be inhibited in neonates to cause a reduction in thyroid hormone synthesis. However, this relationship has not been explored in neonates and is not well understood in adults. The National Research Council perchlorate report (NRC 2005) provided the following estimate: “To cause declines in thyroid hormone production that would have adverse health effects, iodide uptake would most likely have to be reduced by at least 75% for months or longer.” However, to our knowledge, no human or animal data exist that directly support this estimate. Epidemiologic studies in regions of mild deficiency provide indirect estimates of the degree to which iodide must be reduced before adverse consequences occur. Specifically, these studies show that iodine intake that is 40–50% of that recommended by the World Health Organization is associated with adverse consequences in infants and children, including lower IQ and an increased incidence of attention deficit disorder (Aghini Lombardi et al. 1995; Vermiglio et al. 2004). These authors speculate that this association is caused by thyroid hormone insufficiency secondary to moderately low iodine intake. Although the relationship between perchlorate-induced iodide uptake inhibition and thyroid function is still poorly understood, it is likely that the degree of inhibition required to affect hormone status is < 75%. This conclusion is supported by the recent observation that urinary perchlorate levels that are commonplace in the general population are associated with changes in thyroid hormone levels in U.S. women (Blount et al. 2006a).

Insight into the sensitivity of neonates to thyroid hormone insufficiency is perhaps best documented in studies of infants with congenital hypothyroidism (CH) (for review, see Zoeller and Rovet 2004). These studies are particularly useful because subjects are under continuous medical surveillance, so there is good documentation of the relationship between endogenous thyroid hormone, levels of hormone supplementation, and developmental outcome (Heyerdahl and Oerbeck 2003). The neuropsychological outcome of children diagnosed with CH at birth is associated with both the severity of CH and early treatment factors (how soon T_4_ was administered, starting dose and serum T_4_ levels during the first 2 years of life). These T_4_ parameters were highly correlated with verbal IQ at 20 years of age, and children with CH who ultimately completed high school had a significantly higher T_4_ starting dose than those who did not (Oerbeck et al. 2003). Interestingly, the difference in mean starting dose between these two groups was only 2.1 μg/kg/day. Because iodine represents 65% (weight/weight) of T_4_, the amount of iodine associated with that T_4_ difference is only 1.37 μg/kg/day. Others have found that a difference in starting dose of only 12.5 μg/day (8.13 μg/day iodine equivalent or 2.3 μg/kg/day) was associated with a significant difference in full-scale IQ of 11 points (Selva et al. 2002, 2005). Thus, small differences in available thyroid hormone (and the iodine associated with it) during the first few weeks of life can have significant lifetime consequences.

These increased demands for thyroid hormone production in neonates may be compounded because adaptive mechanisms are not as robust. These mechanisms may include negative feedback responses [i.e., thyroid-stimulating hormone (TSH) response to low T_4_], changes in serum binding proteins or iodothyronine transporters, or changes in deiodinases (Zoeller 2005). Thus, a variety of adaptive mechanisms available to adults may not be available to the neonate, causing the neonate to adapt poorly to iodide uptake inhibition. Studies in rats indicate that the ability of the neonate to adapt to low iodide is poor, that compensation appears to be tissue-specific, and that humans are likely to respond in a similar manner (Pedraza et al. 2006). Mild iodide deficiency lowered T_4_ in the absence of an increase in TSH, suggesting that TSH may not be a sensitive index of thyroid hormone status in early life (Pedraza et al. 2006).

In summary, the data needed to perform quantitative risk assessment for perchlorate in neonates are limited. However, there is ample reason to expect the neonatal period to be highly sensitive to perchlorate-induced iodide uptake inhibition. The neonate receives very little thyroid hormone from breast milk and so must depend on the function of its own thyroid gland in the absence of stored hormone. Further, it is confronted with more rapid hormone turnover. This situation is compounded by the vulnerability of brain development to even small deficits in thyroid hormone levels during this period. Impairment of iodide uptake by perchlorate has been described as a precursor effect in adults, largely because of stored hormone and homeostatic mechanisms that can compensate for the perchlorate-induced biochemical perturbation (NRC 2005). The recent CDC data suggest that there may be many women in whom these compensatory mechanisms are inadequate even at background levels of perchlorate (Blount et al. 2006a). The consequences in neonates may be more significant and lead to long-term risks for neurocognitive deficits.

### Lack of epidemiologic studies that assess perchlorate effects in breast-fed infants

Several studies have addressed the association between perchlorate levels in drinking water and thyroid status of the neonate or child (Brechner et al. 2000; Buffler et al. 2006; Crump et al. 2000; Kelsh et al. 2003; Lamm 2003; Lamm and Doemland 1999; Li et al. 2000a, 2000b; Schwartz 2001; Tellez et al. 2005). Most of these studies have failed to find an adverse relationship, although there are a few exceptions. Interpretation of this body of evidence is difficult because the studies suffer from the fact that they were of ecologic design, and because no information is provided on an exposure route of primary concern to neonates, breast-feeding. Regarding limitations due to ecologic design, the levels of perchlorate actually consumed were not known in any of the studies. This has the potential to bias results toward the null, especially in the case of perchlorate, given its prevalence in the diet (El Aribi et al. 2006; FDA 2004). This leads to the potential for exposure misclassification because studies typically categorized exposure simply on the basis of perchlorate levels in a common water supply. This limitation applies to infant exposures that come from breast milk and to post-weaning exposures where perchlorate can come from the child’s diet and drinking water. Although it would not affect studies involving bottle-fed infants during the first months of life, we are not aware of any studies that have specifically evaluated this category of receptor.

None of the studies addressed the exposure under consideration in the present analysis: exposure to the nursing infant through breast milk. In any of the studies it is likely that some infants were breast-fed and others were not. Without this specified, one cannot analyze the relationship between nursing exposure to perchlorate and thyroid status. Several studies performed in the western United States examined the association between perchlorate in drinking water and neonatal thyroid hormones (Brechner et al. 2000; Buffler et al. 2006; Kelsh et al. 2003; Lamm 2003; Lamm and Doemland 1999; Li et al. 2000a, 2000b; Schwartz 2001). This includes three studies that followed infants past the neonatal period: Li et al. (2000a) examined TSH levels at 2–7 and 8–30 days of age in a small subset of children with low T_4_ levels; Li et al. (2000b) examined T_4_ levels in infants as a function of age from day 1 to 60 examined cross-sectionally based on residence in Reno, Nevada (no perchlorate in drinking water), compared with Las Vegas, Nevada (perchlorate in drinking water); and Brechner et al. (2000) examined TSH levels between 0 and 132 days of age in Yuma, Arizona (with perchlorate in the drinking water), versus Flagstaff, Arizona (no perchlorate). The Li et al. studies (2000a, 2000b) did not find an association with perchlorate exposure, whereas the Brechner et al. study (2000) did. Aside from limitations of ecologic design and lack of information on nursing exposure, these studies were limited in other respects. For example, Li et al. (2000a) measured TSH in only a small fraction of infants for whom T_4_ levels were at the low end of the distribution, thereby examining a subsample of infants that was not representative of the population. Additionally, TSH levels were treated as a dichotomous variable based on a definition of clinical disease, even though levels were available for analysis as a continuous variable. The Li et al. (2000b) study of infants out to 2 months of life suggests that levels of perchlorate of up to 15 μg/L in Las Vegas did not affect T_4_ levels. However, the Las Vegas drinking-water perchlorate levels fluctuated widely during this time, so it is difficult to draw conclusions about perchlorate exposure based on city of residence. The Brechner et al. (2000) study has been questioned on the grounds that Flagstaff represents an inappropriate reference location because of its much higher elevation (Lamm 2003).

The series of studies in Chile (Crump et al. 2000; Tellez et al. 2005) were the most detailed but shared the deficiencies and inconsistencies described above for the U.S. studies. Although neonates and first- and second-grade schoolchildren were evaluated, there were no measurements during infancy and no information on breast-feeding exposure. We analyzed these studies as ecologic studies even though biomonitoring data were available in one case (Tellez et al. 2005). However, the biomonitoring data were not used to test associations between perchlorate and hormone status or goiter. There was no evidence for a perchlorate-related difference in TSH, triiodothyronine (T_3_), or T_4_, based on city of residence, but the incidence of goiter in children was greater in the two cities with the higher levels of perchlorate in water. For the history of thyroid disease in the family, the high-perchlorate city (Taltal) had a significant increase compared with the reference city (Antofagasta). The environmental and bio-monitoring data from the Chilean study is described further in “Methods Used in Current Analysis.” As pointed out below, the high iodide intake in these Chilean cities may have affected the outcome of their study.

Overall, the epidemiologic studies do not provide a body of evidence for determining whether perchlorate will affect thyroid status or neurodevelopment in infants. Therefore, the mechanistic and developmental information described in other sections of this article are critical in evaluating whether the postnatal period is likely to be vulnerable to perchlorate.

### Toxicokinetic considerations in the neonate

Perchlorate is cleared unchanged in the urine although protein binding can retain perchlorate in serum and retard its excretion (Clewell et al. 2003; Yu et al. 2002). Biomonitoring studies have capitalized on this excretory pathway because urinary perchlorate is an excellent biomarker for the general public (Blount et al. 2006c). However, there are no data on the efficiency of perchlorate excretion in early life stages in humans and only limited data in rats. In general, human infants have immature renal function and less urinary clearance of many water soluble chemicals (Ginsberg et al. 2002; Kearns and Reed 1989; Morselli 1989). This suggests that slower clearance may be another factor for increased vulnerability to perchlorate. However, data from pre-weanling rats suggest the opposite; rat pups had a higher perchlorate dose than their mothers, but had lower serum concentrations (Clewell et al. 2003; NRC 2005, Appendix E).

The rat data are of questionable relevance to human infants, given the variety of cross-species differences in the ontogeny of toxicokinetic systems (Ginsberg et al. 2004). Regarding perchlorate, cross-species extrapolation of chemical fate is affected by apparent differences in plasma protein binding and renal clearance between rats and adult humans as simulated in well-calibrated toxicokinetic models (Clewell et al. 2003; Merrill et al. 2005). The relevance of the neonatal rat data (Clewell et al. 2003) to human infant dosimetry is also affected by the fact that *a*) rat dams drink nearly all of the urine excreted by their pups, which inflates the serum level of perchlorate relative to the pup; and *b*) lactating dams and pups were dosed with radioactive iodide, which may affect perchlorate toxicokinetics, especially with regard to competition for serum-binding sites. Another uncertainty is the manner in which iodine intake may affect perchlorate toxicokinetics and how this may differ across species and life stages. These uncertainties prevent one from drawing conclusions on the role of perchlorate toxicokinetics to affect dosimetry and risk in human infants.

### Added risk factor: potential lowering of breast-milk iodide

An additional reason to highlight nursing infants as a vulnerable population is that perchlorate risks may be magnified in this group by causing a concomitant decrease in breast-milk iodide levels. The sodium iodide symporter that is expressed in the thyroid gland is also expressed in lactating mammary gland. It transports iodide into breast milk, with perchlorate able to take iodide’s place and be selectively pumped into breast milk (Clewell et al. 2003). This can lead to exposure to perchlorate in nursing infants, while at the same time leading to lower levels of iodide in breast milk. This has been demonstrated in rats where perchlorate exposure to nursing dams resulted in decreased levels of iodide in milk (Clewell et al. 2003). It is expected that this effect on breast-milk iodide will be modified by variations in dietary iodine intake. However, the interaction between perchlorate and iodine ingestion on breast-milk content of iodide has not been studied in rats or humans. Perchlorate may also impair iodine excretion into breast milk in humans, as suggested by data showing an inverse correlation between perchlorate and iodide concentrations in breast milk in a small number of U.S. samples that were > 10 μg/L perchlorate (Kirk et al. 2005). Tellez et al. (2005) did not see a correlation, inverse or otherwise, between perchlorate and iodide concentrations in breast milk across three Chilean cities with widely differing concentrations of perchlorate in drinking water. However, there does seem to be a factor that depresses iodide levels in breast milk in these Chilean cities relative to the United States. On average, Chilean breast-milk iodide concentrations were 40% lower than in U.S. women despite the fact that iodide intake rates are known to be higher in these Chilean cities than in the United States (Kirk et al. 2005; Tellez et al. 2005). The factor responsible for lower-than-expected breast-milk iodide levels in Chile may be that baseline (dietary) exposure to perchlorate is approximately three times higher in Chile than in the United States (Valentin-Blasini et al. 2005).

The reason the Chilean cross-sectional study did not find an inverse correlation between breast-milk levels of perchlorate and iodide is unclear, but comparisons were performed only on the basis of group mean (Tellez et al. 2005). Regression analysis of the entire data set would be a more sensitive method to determine whether there is a significant relationship between these breast-milk parameters in Chile. Further, the greater intake of iodide in Chile may have ameliorated the perchlorate effect on breast-milk iodide. Overall, the potential for perchlorate intake by lactating women to lower the iodide content of breast milk provides additional rationale to consider this life stage to be of prime concern for human risk assessment and standard setting.

## Methods Used in Current Analysis

Calculations of infant exposure to perchlorate involve either fixed inputs, generally the central tendency value, or inputs that take the form of a distribution of values. Monte Carlo simulation analyses are used to present the variability distributions for nursing exposure under baseline conditions (dietary perchlorate only) and with the added exposure associated with the OSWER groundwater cleanup target of 24.5 μg/L. The overall exposure equation is:


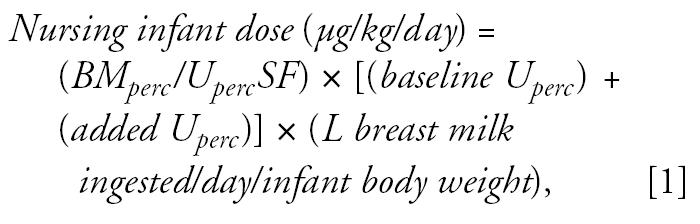


where *BM_perc_/U_perc_SF* is the breast milk perchlorate-to-urinary perchlorate slope factor (micrograms per liter per micrograms per gram creatinine) as derived from the Chilean study (Tellez et al. 2005). *Baseline U_perc_* is the baseline perchlorate excretion in the United States from diet (no drinking water exposure) (micrograms perchlorate/grams creatinine) taken from the National Health and Nutrition Examination Survey (NHANES) 2001–2002 biomonitoring data for 15- to 44-year-old women (Blount et al. 2006c). As described below, this is included in Monte Carlo simulations as a lognormal distribution. *Added U_perc_* is the increase in urinary perchlorate (micrograms per gram creatinine) from tap water ingestion at the OSWER ground-water PRG of 24.5 μg/L. This was calculated as follows:


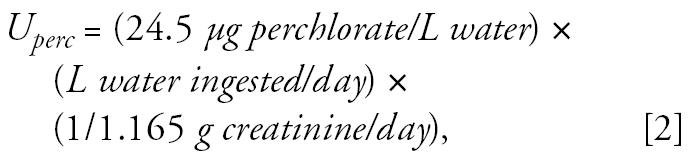


where liters of water ingested by lactating women was entered as a normal distribution with a mean ± SD of 1.189 ± 699 L/day (U.S. EPA 2002); and grams creatinine excretion per day was based on data from 10 women of child-bearing age (Lentner 1981).

*L breast milk ingested-day/infant body weight* is the neonate consumption rate estimated at 2 weeks of age to capture the average over the first month of life; input as a normal distribution with mean 171.8 ± 26.46 mL/kg/day (U.S. EPA 2002).

The following sections provide additional details on the parameters needed to calculate infant exposure to perchlorate via breast milk.

### *Relationship between urinary and breast-milk perchlorate* (BM_perc_/U_perc_SF)

Urinary perchlorate is a reasonable index of the rate of perchlorate intake under conditions of frequent and relatively uniform exposure in which pseudo-steady state toxicokinetics are achieved. The biomonitored populations in Chile are exposed on a daily basis to perchlorate in the diet and drinking water, so it is reasonable to assume that their blood concentration is relatively stable and approaching steady state. The urinary concentration-to-intake dose interconversion is facilitated by the fact that excretion of unchanged parent compound is the main means of elimination. Three different Chilean cities were studied which had a wide range of perchlorate exposure via drinking water, as shown in Table 1 (Tellez et al. 2005). This table also shows the urinary and breast-milk perchlorate data for these cities. The breast-milk statistics for Antofagasta do not include one outlier subject who had very high breast-milk perchlorate (1,042 μg/L).

We used linear regression to relate the mean concentrations of perchlorate in breast milk to urine across the three cities. The regression line was weighted by the inverse of the variance in the data for each city. The line was forced through the origin because it can be expected that with zero intake by the mother (and thus no perchlorate in urine), there should also be none in breast milk. The size of the sampled groups differed considerably between the urinary and breast-milk measurements (Table 1). The lack of paired measurements from the same individuals precludes a more precise analysis of the correlation between these parameters.

### *Baseline urinary perchlorate concentration in the U.S. population without drinking water exposure* (baseline U_perc_)

**Figure 1:**
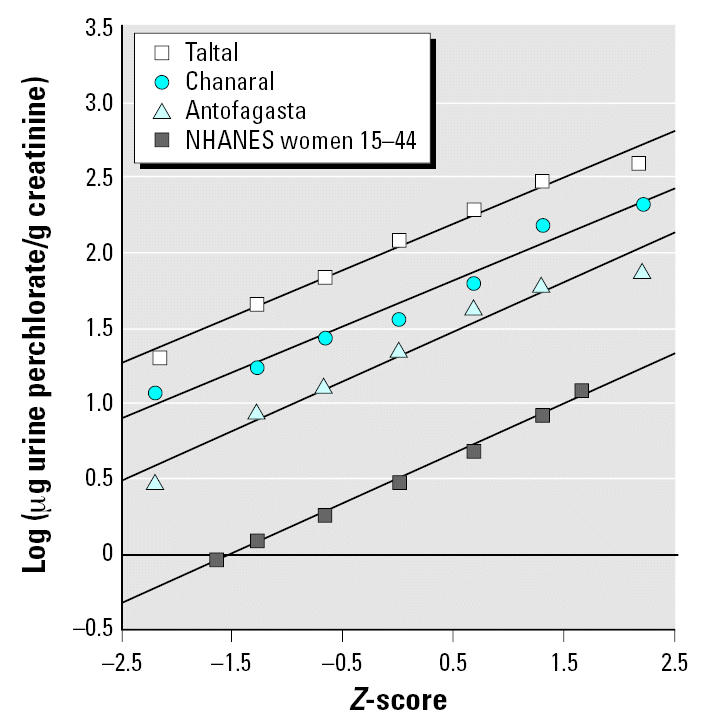
Log-normal probability plots of the distributions of urinary perchlorate excretion in three Chilean cities and the United States. Taltal: *y* = 2.03 + 0.306*x; R*^2^ = 0.984. Chanaral: *y* = 1.66 + 0.304*x; R*^2^ = 0.968. Antofagasta: *y* = 1.31 + 0.326*x; R*^2^ = 0.962. NHANES: *y* =0.50 + 0.332*x; R*^2^ = 0.997.

We assessed urinary perchlorate concentrations based on a randomized sample (*n* = 2,818) intended to be representative of the U.S. general population ≥ 6 years of age, as part of the NHANES 2001–2002 biomonitoring campaign (Blount et al. 2006c). This is a large expansion on the earlier perchlorate biomonitoring data set published by the CDC from a convenience sample of 61 adult residents of Atlanta, Georgia (Valentin-Blasini et al. 2005). The larger data set includes results for 662 women, 15–44 years of age. This subsample was selected as the baseline population to evaluate nursing exposures. Although a baseline population of nursing mothers would have been ideal, NHANES did not obtain a sizeable or representative sample from this group. The urinary data for the sample of 662 women as well as the urine biomonitoring data from the Chilean cities appear to be log-normally distributed as evidenced by the fit of the data to regression lines in log probability plots for each city (Figure 1) (for background on probability plots, see Hattis and Burmaster 1994). Therefore, dietary perchlorate intake inferred from urinary perchlorate data from Atlanta were input as a log-normal distribution to represent baseline perchlorate exposure for Monte Carlo analysis.

**Figure 2:**
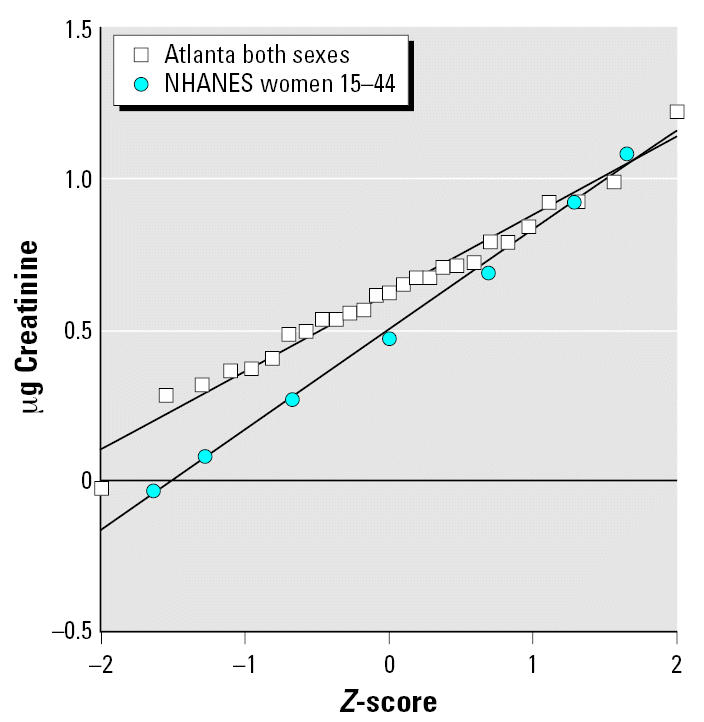
Comparison of urinary perchlorate (μg/g creatinine) between Atlanta sample of 27 adults versus national sample of women 15–44 years of age. Atlanta: *y* = 0.622 + 0.258*x; R*^2^ = 0.974. NHANES: *y* = 0.498 + 0.332*x; R*^2^ = 0.997.

The group sampled by NHANES is assumed to represent baseline perchlorate exposure that comes from the diet without a substantial contribution from drinking water. NHANES did not obtain drinking-water perchlorate data. However, a small pilot study conducted by the CDC measured urinary perchlorate in conjunction with drinking-water perchlorate and dietary factors for 27 subjects in Atlanta (Blount et al. 2006b). Drinking-water perchlorate averaged only 0.11 μg/L (range < 0.05–0.25 μg/L) for these 27 individuals, a small contribution given that these subjects were excreting approximately 5 μg perchlorate/day. The importance of diet in determining urinary perchlorate was shown by dividing the group into those who ate one or fewer versus three or more servings of dairy or leafy green vegetables. The higher servings group had an average urinary perchlorate that was 1.8-fold higher. Figure 2 shows urinary perchlorate for all 27 Atlanta subjects compared with the 662 women sampled by the NHANES 2001–2002. The log probability plot shows a reasonable correspondence between these data sets. The Atlanta sample had a higher median but the two populations converged at the upper end of the distribution. Because the Atlanta distribution was not materially affected by drinking-water intake, Figure 2 suggests that the NHANES distribution, even at the upper end, is what can be expected from diet alone. Therefore, we considered the NHANES urinary perchlorate distribution for these women as a reasonable baseline for projecting the added impact of drinking water at the OSWER PRG.

We modeled the contribution of maternal water ingestion to urinary perchlorate by combining the expected perchlorate exposure associated with a normal distribution of daily water consumption with the log-normal distribution of baseline urinary perchlorate. Correction was made for creatinine excretion rate per day as described above. These equations also assume that perchlorate ingested per day is equal to perchlorate urinary excretion per day under pseudo-steady state conditions. The Monte Carlo simulations were done in triplicate runs of 5,000 trials each.

## Results

### Evaluation of the impact of the OSWER groundwater PRG on exposures and risks to nursing infants

**Figure 3:**
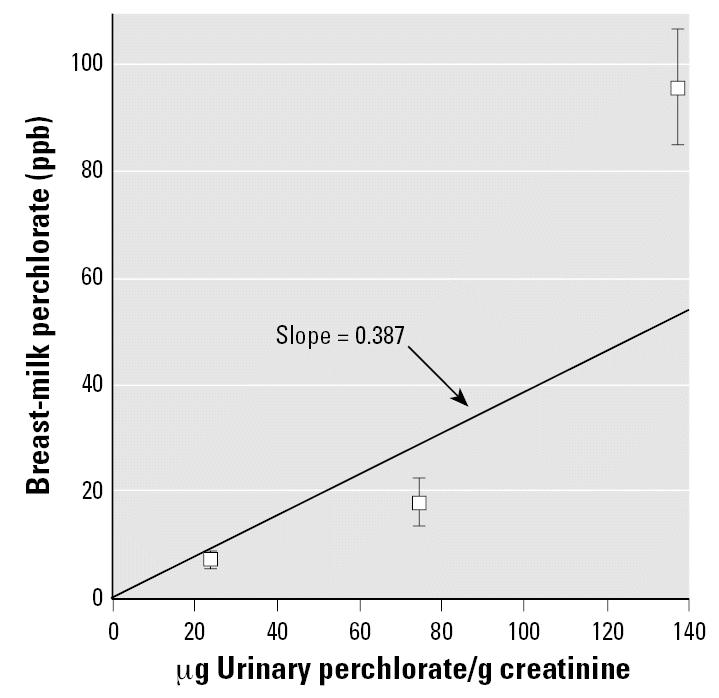
Mean (± SD) breast-milk perchlorate concentration in relation to mean urine perchlorate excretion. Data adapted from Tellez et al. (2005). Inverse invariance-weighted straight line constrained to pass through the origin.

The relationship between perchlorate exposure, as estimated from urinary perchlorate excretion, and perchlorate in breast milk is shown in Figure 3 across the three Chilean cities. This is the only data set that provides both parameters for the same population. The regression line for these three cities (weighted by inverse variance of the mean of each datapoint) provides a slope of 0.387 (micrograms per liter breast milk per micrograms per gram creatinine). The line is less influenced by the Taltal results than by the other data points because of the large uncertainty in the mean breast-milk concentration for the Taltal data.

**Figure 4:**
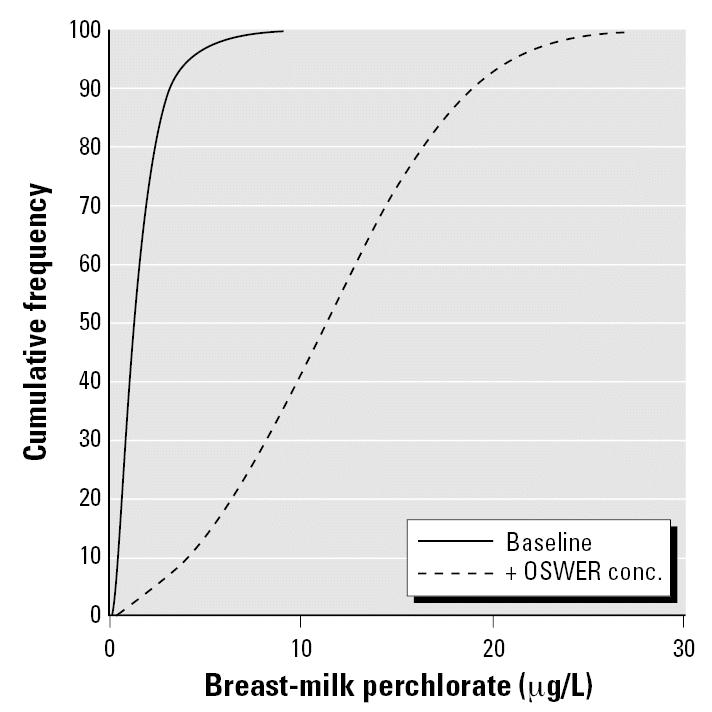
Simulated cumulative distribution of breast-milk concentrations for baseline and OSWER PRG scenarios.

We used this slope to convert urinary perchlorate to breast-milk perchlorate for the baseline U.S. population distribution as derived from the 668 women sampled by NHANES. The distribution of breast-milk perchlorate in this baseline population is shown in Figure 4, which has a simulated mean ± SD of 1.63 ± 1.5 μg/L. The vast majority of the baseline population (> 90%) would be expected to have measureable perchlorate in breast milk at a detection limit of 0.4 μg/L (detection limit from Kirk et al. 2005). This agrees with the high rate of detection seen in the limited breast-milk data currently available (Kirk et al. 2005). Figure 4 also shows the total perchlorate in breast milk after adding to the baseline a daily tap water exposure of 24.5 μg/L (the OSWER PRG). The distribution is shifted approximately 7-fold to the right with a new mean ± SD of 11.45 ± 5.7. The 99th percentile value is 25.2 μg/L.

**Figure 5:**
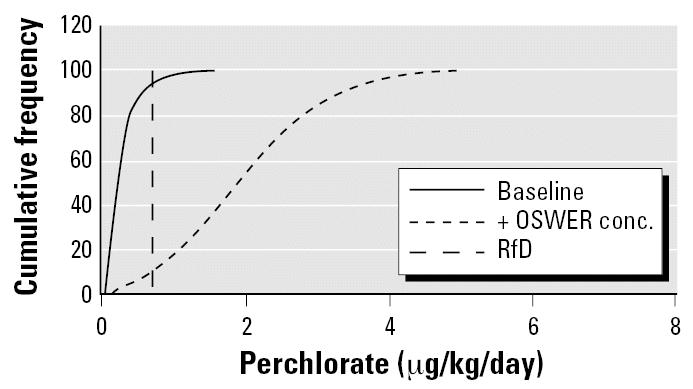
Daily doses from nursing infant exposure to perchlorate under baseline and (+) drinking water scenarios.

We then used these breast-milk concentrations to simulate nursing exposure in infants within the first month of life, as represented by the intake rate per body weight for 2-week-old infants. The resulting exposure dose for both the baseline and (+) OSWER (an added 24.5 μg/L in drinking water) scenarios is presented in Figure 5. The two distributions are quite distinct, with the log-normal baseline distribution overlapping only about 30% of the (+) drinking water distribution. Figure 5 also shows the U.S. EPA/Integrated Risk Information System (IRIS) RfD (0.7 μg/kg/day). The RfD is surpassed at approximately the 95th percentile of the baseline population and at the 15th percentile of the (+) drinking water distribution. The average nursing infant exposure dose for the baseline population is 40% of the RfD, whereas the average for the (+) 24.5 μg/L drinking-water scenario is 2.8-fold greater than the RfD. The 95th percentile of the (+) drinking-water distribution exceeds the RfD by 5.4-fold. These results suggest that perchlorate exposure associated with the OSWER PRG, in conjunction with background dietary exposure, results in exposures to nursing infants in excess of the RfD for the great majority (85%) of the population. In fact, the upper end of the baseline distribution is also above the RfD.

Table 2 shows the baseline distribution of nursing infant exposure as projected from the NHANES data set, along with the perchlorate drinking-water targets that would satisfy the RfD at different percentiles of the distribution. Adherence to the RfD would require a groundwater cleanup level of 6.9 μg/L for the 50th percentile of the baseline distribution, and 1.3 μg/L at the 90th percentile.

### Evaluation of the RSC needed to protect in utero development and nursing infants

A key consideration in setting the PRG is whether an RSC term is needed to protect sensitive life stages (*in utero*, postnatal) and what the value should be. This depends on the extent of non-drinking-water exposure to perchlorate relative to the perchlorate RfD. In this section we use the NHANES data for 668 women to provide an indication of dietary contribution to perchlorate exposure for RSC consideration.

Table 3 shows the daily exposure dose in adults implied by the urinary excretion data from NHANES (Blount et al. 2006c). When central estimates from the NHANES study are considered, dietary exposure appears to constitute approximately 10% of the RfD. At the 95th percentile, diet is still only 32% of the RfD, which would support an RSC of 0.7 in the case of pregnant women. This is considerably larger than the default RSC of 0.2 commonly used in drinking-water risk assessments.

These estimates pertain to adult baseline (dietary) exposure as a percentage of the RfD. Review of Figure 5 indicates that baseline exposure of nursing infants is 40% of the RfD for average exposure and it exceeds the RfD at the 95th percentile. Therefore, baseline dietary exposure of the mother produces a nursing infant–based RSC in the range of 0.6 (average case) to 0 (no allowance for drinking water exposure). This latter estimate of the nursing infant–based RSC is obtained because the RfD is exceeded at the 95th percentile of exposure in the maternal diet-to-breast-milk-to-nursing infant pathway.

## Discussion

The OSWER PRG is an important guidance for the Superfund program in that it establishes the initial groundwater target that, if surpassed, would attract the attention of site managers and health officials. It is not necessarily the final cleanup level because it can be increased or decreased based on site-specific considerations. The factors presented in this article have little to do with site-specific features but rather address exposure and toxicity issues relevant to all sites where there is potential for groundwater ingestion by pregnant women, nursing mothers, and infants. The higher dose rate received by nursing infants and the contribution of diet to total perchlorate dose are key considerations, and represent an opportunity for improving the scope and public health protectiveness of the PRG.

Our literature review and analysis indicate that there are toxicodynamic and toxicokinetic reasons to consider early postnatal life as a particularly vulnerable time for perchlorate toxicity. Further, this life stage has not been adequately assessed in perchlorate epidemiology studies. This is a critical issue because, as suggested elsewhere (Baier-Anderson et al. 2006; Kirk et al. 2005) and as presented in this article, infants can have greater perchlorate exposure than people at other life stages from ingestion of reconstituted formula or breast milk. This provides an imperative to evaluate nursing infants in risk assessments of perchlorate in drinking water.

In the current analysis we incorporate data on urinary and breast-milk perchlorate concentrations into a Monte Carlo analysis of the distribution of intake of perchlorate by nursing infants at the OSWER PRG. Our simulations indicate that 85% of nursing infants can be expected to exceed the RfD, with the average exceedance 2.8-fold. In fact, the perchlorate drinking-water concentration needs to be < 6.9 μg/L to keep the 50th percentile nursing infant below the RfD. The corresponding value for the 90th percentile infant is 1.3 μg/L. These drinking-water concentrations are 4- to 19-fold below the OSWER PRG and are more in line with proposals for regulating perchlorate in groundwater in a number of states (MADEP 2006; New Jersey Drinking Water Quality Institute 2005; Ting et al. 2006). Ideally, one would develop a perchlorate groundwater target that keeps 95% of nursing infants below the RfD, but this would require a target of < 1 μg/L. Comparison of Tables 2 and 3 shows that perchlorate doses are expected to be approximately 3-fold higher in nursing infants than in adult women.

Use of biomonitoring data to evaluate the RSC in adult women found that dietary perchlorate likely represents 32% of the RfD when considering the 95th percentile of the NHANES distribution. This corresponds to an RSC of 0.7, which is somewhat higher than the RSC applied by the California EPA (0.6) (Ting et al. 2006). However, the RSC would be in the range of 0.6 to 0 when considering the baseline exposure of nursing infants from mother’s diet-only intake of perchlorate. Thus, protection of nursing infants from perchlorate would require an RSC at least as low as the default often used in setting drinking-water standards—0.2. The lack of an RSC in OSWER’s PRG derivation in effect assumes that 100% of the daily perchlorate exposure comes from drinking water, omitting the contribution from diet. Thus, there is considerable room for reevaluation of the PRG. The RSC estimate is based on a particular RfD. Obviously, if the RfD were lower, then the baseline (dietary) exposure would constitute a larger fraction, necessitating a decrease in the RSC. For example, using benchmark dose analysis from the Greer et al. (2002) data set, the California EPA derived a perchlorate toxicity point of departure that is approximately 2-fold lower than that used in the IRIS RfD (Ting et al. 2006). Based on this toxicity value, the RSC estimate would need to be cut in half. Further, recent evidence from the same NHANES/CDC data set described above indicates an effect on thyroid hormone status in adult women at perchlorate intake levels that are below the RfD (Blount et al. 2006a). Table 3 shows the average intake of women in the NHANES study to be approximately 10-fold below the IRIS RfD. This is likely in the range of perchlorate effect levels, given that Blount et al. (2006a) found the thyroid hormone effect along a continuous function with perchlorate dose that spanned the center of the exposure distribution. Therefore, future evaluations of the RSC will need to take into account any changes in the RfD that may occur as the human dose-response is reanalyzed.

### Uncertainties

The current analysis requires knowledge of baseline exposure to perchlorate via nondrinking water sources, primarily the diet. The recent biomonitoring data set developed as part of NHANES 2001–2002 (Blount et al. 2006c) provides a very useful starting point for estimating the population distribution of dietary perchlorate intake. The comparison against biomonitoring results from 27 Atlanta residents who had minimal perchlorate in their tap water (Figure 2) indicates that the NHANES distribution is likely a reasonable estimation of background (dietary) exposure. What is less certain is the conversion of the urinary biomonitoring level to intake dose for these subjects. This is a key starting point for perchlorate risk assessment. The central assumption is that the amount of perchlorate excreted per day equals the amount ingested in the biomonitored individuals. This is true if these individuals are near or at steady state. In such cases the daily exposure can be viewed as a maintenance dose which keeps body stores at a relatively constant level i.e., no net accumulation or loss. This occurs in people whose exposure rate is fairly uniform, a situation that can be expected for perchlorate because it is present in a variety of foods (El Aribi et al. 2006; FDA 2004). This approach for relating urinary bio-monitoring data and intake rate has also been used for other chemicals at or near steady state, such as phthalates (Koch et al. 2003; Kohn et al. 2000; Koo et al. 2002) and chlorpyrifos (Rigas et al. 2001).

A caveat with this application of biomonitoring data is that it depends on urinary excretion data normalized per gram of creatinine, which is multiplied by the creatinine excretion rate per day to yield the daily perchlorate excretion rate. However, the creatinine excretion rate per day is not typically measured in individual subjects; rather, a central population estimate is used, as in the present analysis. This does not account for the considerable interindividual variability in creatinine excretion as seen in an analysis of NHANES III data (Barr et al. 2005). This source of variability is not expected to bias the analysis in a particular direction, but could be incorporated into future Monte Carlo analyses by including the creatinine excretion rate as a distribution rather than a fixed central estimate.

It is useful to contrast estimates of perchlorate dietary ingestion in the NHANES data set with those developed elsewhere. Data from the three Chilean cities suggest that dietary perchlorate is consistent across the three cities, 20–35 μg/day on average, or 0.3–0.5 μg/kg/day (Tellez et al. 2005). This is approximately 5-fold above the average intake rate we calculated for 15- to 44-year-old women sampled by NHANES—0.075 μg/kg/day (Table 3). This is consistent with there being higher perchlorate in soil, fertilizer, and locally grown foods in Chile than in the United States (Crump et al. 2000; El Aribi et al. 2006).

Another area of uncertainty is the conversion of biomonitored levels of perchlorate in urine to breast milk perchlorate. We used the only available data set that provides both urinary and breast-milk biomonitoring data, the Chilean data from three cities (Tellez et al. 2005). The strength of this data set is that it captures a wide range of perchlorate exposures. However, the number of subjects for which breast-milk data were available is considerably smaller than the number of subjects for which urinary data were available. The lack of pair-matched results meant that the correlation could be determined only on a population mean basis, relying on only three data points, one for each city, rather than the individual data points. Although the line in Figure 3 represents a reasonable best fit across the three cities, a more robust and comprehensive analysis would have been possible if individual, pair-matched data were available.

**Figure 6:**
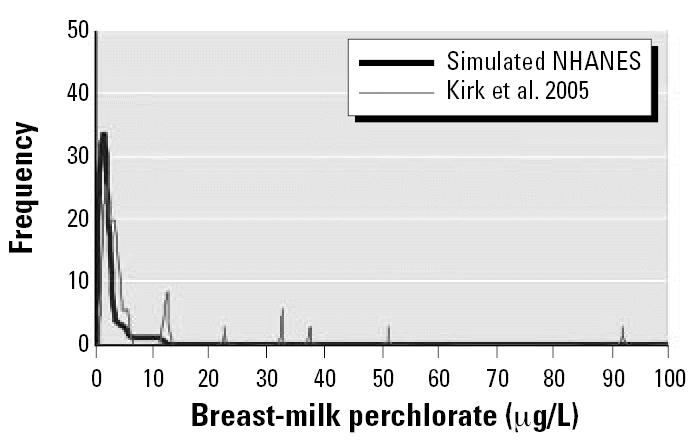
Breast-milk perchlorate simulated from NHANES data versus actual data. Reported by Kirk et al. (2005).

A potentially greater uncertainty in using the Chilean data to represent the urinary-to-breast-milk relationship is that this relationship may be different in the United States. Although iodide supplementation in these Chilean cities has decreased in recent years, iodine intake in Chile still appears to be higher than in the United States (Tellez et al. 2005). This may affect, via substrate competition, the distribution of perchlorate into various compartments into which it is actively transported. Just as high perchlorate impairs iodide excretion into breast milk, it is also possible that high iodide decreases perchlorate entry into breast milk. This would cause a shallower breast-milk-to-urinary-perchlorate slope in Figure 3 and underpredict nursing infant exposures in the United States. That this may be the case is presented in Figure 6, which compares breast-milk perchlorate simulated from the NHANES data with actual data from Kirk et al. (2005). The actual data were collected from 36 women across 18 states in the United States. Both distributions appear log-normal with the bulk of the results < 10 μg/L. However, the Kirk et al. (2005) distribution is shifted to the right of the NHANES-based simulation (Kirk et al. median = 3.3 μg/L; NHANES simulation median = 1.2), and there are numerous high-end individuals in the Kirk et al. data set not predicted by the simulation. The higher perchlorate levels in the Kirk et al. data set may be caused by greater perchlorate intake from the diet or drinking water than in the NHANES subjects, although there is no reason to think that the Kirk et al. population was biased toward high-exposure individuals. A distinct possibility is that the slope between breast-milk and urinary perchlorate is greater in the United States than in Chile, causing our simulations of nursing exposure and risk to be an underestimate. This would also lower the OSWER groundwater PRG needed to keep nursing infants below the current RfD. More studies are needed that define perchlorate levels in U.S. breast milk and that explore the interaction between iodide and perchlorate at the mammary symporter.

## Conclusions

The neonate may be particularly vulnerable to perchlorate toxicity because of a number of factors described in this article. This means that the OSWER PRG of 24.5 μg/L should be evaluated in light of neonatal exposures. Baseline exposure as simulated from NHANES biomonitoring data takes up a substantial fraction of the RfD in nursing infants, which does not allow much additional perchlorate exposure from drinking water. In this regard, the OSWER PRG could result in perchlorate exposures that exceed the RfD in a high percentage of nursing infants. This is generally true for pregnant women because an RSC was not used in OSWER’s PRG calculation. Proposed drinking-water standards set for perchlorate by several states (New Jersey, Massachusetts, California) are in the range of 2–6 μg/L, well below the OSWER PRG. We recommend that OSWER reevaluate the perchlorate PRG in light of the early-life exposure and RSC factors raised here. In addition. recent data from the CDC on perchlorate’s effects on thyroid status in adult women (Blount et al. 2006a) need to spur follow-up studies and become incorporated into future perchlorate risk assessments.

## Figures and Tables

**Table 1:** Biomonitoring results (mean ± SD) for three Chilean cities.

	Antofagasta	Chanaral	Taltal
Tap water perchlorate (μg/L)	ND (< 4)	5.82 ± 0.63	114 ± 13.3
Urinary perchlorate (μg/g creatinine)	28.4 ± 22	80.2 ± 129.6	135.5 ± 95
No.	61	53	59
Breast-milk perchlorate (μg/L)	7.7 ± 7.5^a^	18.3 ± 17.7	95.6 ± 54.6
No.	13	16	25

Data adapted from Tellez et al. (2005). ND, not detected.

a The Antofagasta breast-milk data reflect one less sample than reported by Tellez et al. (2005) due to an outlier in this group. The mean was recalculated by multiplying the original mean (81.6 μg/L) by the original no. (14), subtracting the outlier (1,042 μg/L) and then dividing by the new no. (13). Variability in this group was assumed to be on a par with that in Chanaral.

**Table 2:** Drinking-water targets for different percentiles of the perchlorate exposure distribution.^a^

Percentile	Baseline nursing infant exposure (μg/kg/day)	Drinking-water target (μg/L) to maintain infant at RfD
0.5	0.028	> 24.5
1	0.034	> 24.5
2	0.042	> 24.5
5	0.058	> 24.5
10	0.076	> 24.5
25	0.122	12.4
50	0.206	6.9
75	0.347	3.8
90	0.562	1.3
95	0.744	—b

a Baseline distribution is that derived for the NHANES data set. This is overlaid with the distribution of maternal exposure to perchlorate and transfer to nursing infant.

b There is no drinking-water concentration that can satisfy this condition because the baseline exposure is already above the RfD for a nursing infant.

**Table 3:** Percentage of the IRIS RfD taken up by non-drinking-water sources in 15- to 44-year-old women sampled in NHANES 2001–2002.^a^

NHANES sample	Urinary output (μg/g creatinine)	Maternal dose (μg/kg/day)^b^	Percent RfD^c^
50th percentile	2.97	0.056	8
Average	4.0	0.75	11
90th percentile	8.4	0.16	23
95th percentile	12.1	0.23	32

a Urinary perchlorate data adapted from Blount et al. (2006c).

b Converted from perchlorate in urine based on daily creatinine excretion of 1.165 g and adult female body weight of 62 kg.

c IRIS RfD established in 2005 is 0.7 μg/kg/day.
